# Identification and Functional Characterization of *CsMYCs* in Cucumber Glandular Trichome Development

**DOI:** 10.3390/ijms24076435

**Published:** 2023-03-29

**Authors:** Zhongxuan Feng, Lei Sun, Mingming Dong, Shanshan Fan, Kexin Shi, Yixin Qu, Liyan Zhu, Jinfeng Shi, Wujun Wang, Yihan Liu, Xiaofeng Chen, Yiqun Weng, Xingwang Liu, Huazhong Ren

**Affiliations:** 1 Department of Vegetable Science, College of Horticulture, China Agricultural University, Beijing 100193, China; 2 Yantai Institute, China Agricultural University, Yantai 264670, China; 3 USDA-ARS, Vegetable Crops Research Unit, Horticulture Department, University of Wisconsin, 1575 Linden Drive, Madison, WI 53706, USA; 4 Sanya Institute, China Agricultural University, Sanya 572019, China; 5 Engineering Research Center of Breeding and Propagation of Horticultural Crops, Ministry on Education, College of Horticulture, China Agricultural University, Beijing 100193, China

**Keywords:** bHLH, MYC, glandular trichome, cucumber, VIGS

## Abstract

Glandular trichomes (GTs), specialized structures formed by the differentiation of plant epidermal cells, are known to play important roles in the resistance of plants to external biotic and abiotic stresses. These structures are capable of storing and secreting secondary metabolites, which often have important agricultural and medicinal values. In order to better understand the molecular developmental mechanisms of GTs, studies have been conducted in a variety of crops, including tomato (*Solanum lycopersicum*), sweetworm (*Artemisia annua*), and cotton (*Gossypium hirsutum*). The MYC transcription factor of the basic helix-loop-helix (bHLH) transcription factor family has been found to play an important role in GT development. In this study, a total of 13 cucumber MYC transcription factors were identified in the cucumber (*Cucumis sativus* L.) genome. After performing phylogenetic analyses and conserved motifs on the 13 *CsMYCs* in comparison to previously reported MYC transcription factors that regulate trichome development, seven candidate MYC transcription factors were selected. Through virus-induced gene silencing (VIGS), *CsMYC2* is found to negatively regulate GT formation while *CsMYC4*, *CsMYC5*, *CsMYC6*, *CsMYC7*, and *CsMYC8* are found to positively regulate GT formation. Furthermore, the two master effector genes, *CsMYC2* and *CsMYC7*, are observed to have similar expression patterns indicating that they co-regulate the balance of GT development in an antagonistic way.

## 1. Introduction

Trichomes on the aerial parts of plants are usually divided into two types: glandular tichomes (GTs) and non-glandular trichomes (NGTs) [1]. Known as the ‘plant biofactory’ due to their ability to synthesize and store specific secondary metabolites, the GTs structure and the products they synthesize and secrete are agriculturally important for plant adaptation and resistance to external stresses [2,3]. In addition, the metabolites secreted by some specific plant GTs can be used to make medicines and essential oils, such as artemisinin in the GTs of *Artemisia annua* and cannabinoid in the GTs of *Cannabis sativa* L., which have medicinal and economic value [4,5,6]. Therefore, the study of the developmental mechanism of GTs and the synthetic mechanism of their internal metabolites is important for the development of natural products metabolic engineering. Research on the development of GTs and their internal metabolite synthetic mechanism is relatively lagging behind due to the fact that the model plant *Arabidopsis thaliana* only has NGTs. Currently, researchers who study GT development typically focus on horticultural crops such as tomato (*Solanum lycopersicum*) and sweetworm (*Artemisia annua*) [7].

GTs typically consist of a head that secretes secondary metabolites, a stem that supports the head, and a base that connects the stem to the epidermal cells. Based on this structure, GTs are usually divided into two types: peltate and capitate. Peltate GTs have a large, multicellular head and a shorter, unicellular, or multicellular stem, while capitate GTs have a small secretory gland head and a longer, multicellular stem [8]. Cucumber has two types of GTs (type I, type VI), of which the most numerous and widely distributed is type I GTs. Type I GTs are widely distributed on various tissue parts of cucumber, and are a kind of peltate GTs consisting of a four-celled secretory gland head and a shorter multi-celled stem [9].

Cucumber GT development is divided into five stages, namely initiation stage (Stage I), first division stage (Stage II), glandular head transition stage (Stage III), glandular head formation stage (Stage IV), and active metabolism stage (Stage V) [10]. Only a few genes related to the development of cucumber GTs have been reported. Mutation of *CsGL3/CsTril*, encoding an HD-Zip IV transcription factor, causes cucumber to exhibit an overall hairless phenotype with neither GTs nor NGTs [11,12]. Loss of function of *CsTBH/CsGL1/Mict*, encoding an HD-Zip I transcription factor, results in a papillate trichomes phenotype which makes it difficult to distinguish GTs from NGTs [13,14,15,16]. Additionally, the density of cucumber GTs is also regulated by *CsTTG1* which encodes a WD repeat protein [17]. The above genes function in both GT and NGT development in cucumber. However, few studies focus on genes that regulate only GT development in cucumber. In a previous study, virus-induced gene silencing (VIGS) of *CsbHLH1*, encoding a bHLH transcription factor, resulted in a significant decrease in the number of GTs in cucumber leaves [10].

The bHLH transcription factors (TFs) family is one of the largest families of TFs in plants and is involved in various growth and developmental pathways [18,19,20]. The MYC type transcription factor is an important member of the bHLH transcription factor family. The first MYC transcription factor was identified in maize (*Zea mays*) in 1989 [21] and has since been cloned and analyzed in *Arabidopsis* (*Arabidopsis thaliana*), rice (*Oryza sativa*), and wheat (*Triticum aestivum*) [22,23]. With the helix-loop-helix (HLH) structural domain typical of the bHLH TFs, MYCs also have a conserved bHLH-MYC_N domain at the N-terminal, which is associated with DNA binding and plays a role in regulating metabolic and hormonal pathways [24]. Besides, the HLH domain of MYCs can bind to the E-box (CANNTG) or G-box (CACGTG) of the downstream target genes to regulate their expression [25]. However, the MYC-type transcription factors on cucumber have not been systematically studied.

In previous studies, the *Arabidopsis* bHLH family has been divided into 12 subfamilies with thirteen MYCs belonging to subfamily III [26]. The subfamily III is divided into six subgroups, of which the comprehensively studied MYCs belong to IIId, IIIe, and IIIf. The IIIe MYCs *AtMYC2*, *AtMYC3*, *AtMYC4*, and *AtMYC5* play an important role in stress response induced by jasmonic acid (JA), while the IIId MYCs *bHLH3/JAM3*, *bHLH13/JAM2*, and *bHLH17/JAM1* bind DNA with similar specificity to that of *AtMYC2*, *AtMYC3*, and *AtMYC4*, but lack a conserved activation domain [24,27,28,29,30]. The IIId and IIIe subgroups compete for the same cis-regulatory elements of downstream targeted genes to regulate stress response induced by JA [27,28,31,32]. As for the MYCs in IIIf, previous studies have shown that *AtTT8*, *AtMYC1*, *AtGL3*, and *AtEGL3* could regulate the formation of NGTs [33,34,35,36]. In addition to regulating NGT development, MYCs also take part in the development of plant GTs [37]. Tomato MYC-type TF *SlMYC1* plays an important role in glandular cell division and expansion in type VI trichomes to regulate the morphogenesis of tomato GTs [38,39]. Cotton MYC-type TFs *GoPGF* and *GhCGF1* regulate the formation of cotton GTs [40,41]. It has not been reported whether MYC-type transcription factors can regulate the formation of GTs in cucumber.

Here, 13 MYC transcription factors were identified and described in cucumber. Seven genes possibly related to the development of cucumber GTs were screened by phylogenetic analysis and conserved motif analysis. The functions of these seven genes were preliminarily verified by VIGS. The results reveal that *CsMYC2* negatively regulates cucumber GT formation and *CsMYC4,5,6,7,8* redundantly positively regulate cucumber GT formation, among which is the most energetic phenotype of *CsMYC7*. Further expression analysis of *CsMYC2* and *CsMYC7* reveals that they show a similar expression pattern and may regulate the cucumber GT formation in an antagonistic manner.

## 2. Results

### 2.1. Genome-Wide Identification and Phylogenetic Analysis of Cucumber MYC Transcription Factors

To identify MYC transcription factors in the cucumber genome, we downloaded the genome protein sequences through the cucurbit genome website (http://cucurbitgenomics.org/ (accessed on 25 January 2023)) and searched for genes with conserved the bHLH-MYC_N structural domain (PF14215) of MYC transcription factors and obtained a total of 18 candidate genes (Table S1). Among the 18 candidate genes, a total of 13 MYC genes contain both bHLH-MYC_N domain and bHLH domain and were identified in cucumber through the NCBI-CDD database and SMART database, namely *CsMYC1-13* (Table 1). The physical features of these MYC TFs were predicted: The protein length varies from 322 (CsMYC6) to 959 (CsMYC13) amino acids; the pI (Isoelectric Point) varies from 5.11 (CsMYC2) to 8.66 (CsMYC4); and the molecular weight varies from 36.38 kDa (CsMYC6) to 104.48 kDa (CsMYC13) (Table 1). To gain a preliminary understanding of these genes, we looked for their homologs on *Arabidopsis Thaliana*. The homolog of *CsMYC1* is *AtGL3*, which regulates the development of unicellular non-glandular trichomes on Arabidopsis [35] (Table 1). The homolog of *CsMYC2*, *CsMYC3*, and *CsMYC6* is *AtMYC2*, the homolog of *CsMYC4* and *CsMYC5* is *AtbHLH14*, the homolog of *CsMYC7* is *AtbHLH13*, and the homologue of *CsMYC8* is *AtbHLH3* (Table 1). *AtMYC2* encodes a MYC-related transcriptional activator with a typical DNA binding domain of a basic helix-loop-helix leucine zipper motif regulating diverse JA-dependent functions [42]. *AtbHLH13* functions redundantly with *AtbHLH3* and *AtbHLH14*, interacting with JAZ proteins, and negatively regulating jasmonate responses [27,28]. The homolog of *CsMYC9* is *AtAMS*, which is involved in tapetal cell development [43]. *AtEMB1444* and *AtLHW*, the homolog of *CsMYC10*, *CsMYC11*, *CsMYC12*, and *CsMYC13*, which take part in the production of stele cells in root meristems, are required to establish and maintain the normal vascular cell number and pattern in primary and lateral roots [44].

**Figure 1:**
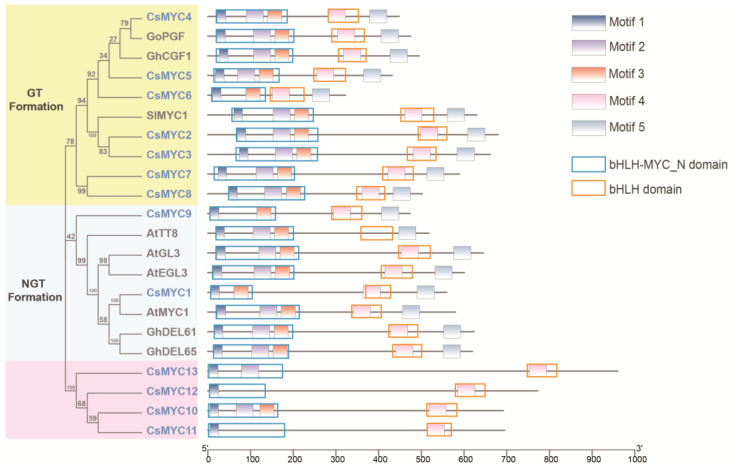
Phylogenetic analysis and conserved motifs analysis of cucumber MYC genes with previous reported-MYCs. Left panel: Molecular phylogenetic analysis of cucumber MYC genes and previous reported-MYCs by maximum likelihood method. Blue text represents CsMYCs. Middle panel: the conserved motifs of MYCs are represented by different colored boxes, the conserved domains of MYCs are represented by different colored rectangle. Cs, *Cucumis Sativus*; Go/Gh, *Gossypium hirsutum*; Sl, *Solanum lycopersicum*; At, *Arabidopsis thaliana*.

To further investigate the function of these MYC genes in trichome development, we performed the phylogenetic clustering analysis by comparing them with the previously reported glandular trichome development-related MYC TFs SlMYC1 [38], GoPGF [40], GhCGF1 [41] and the non-glandular trichome development-related MYC TFs AtGL3 [35], AtEGL3 [36], AtMYC1 [33], AtTT8 [34], GhDEL61 [45], GhDEL65 [46] (Table 2 and Table S2). The tree with the highest log likelihood (−8624.0776) is shown (Figure 1 and Figure S1). The percentage of trees in which the associated taxa clustered together is shown next to the branches. The phylogenetic analysis grouped these genes into three evolutionary branches, *CsMYC2-8* belonging to the evolutionary branch related to GT development, *CsMYC1* and *CsMYC9* belonging to the evolutionary branch related to NGT development, and the other four *CsMYCs* in a new evolutionary branch (Figure 1). Besides, the MEME analysis and NCBI-CCD analysis were carried out to predict the conserved motifs protein sequence and conserved domain to better understand the conservation and diversification of these MYCs (Figure 1). A total five motifs were identified through all the MYC genes. Motif 1, motif 2, and motif 3 are located in the bHLH-MYC_N domain, while motif 4 is located in the bHLH domain. Previous studies have shown that the bHLH-MYC_N domain functions in metabolites biosynthesis and responds to jasmonic acid, while the bHLH domain functions in DNA binding or the dimer interface. A single amino acid change on motif 3 located in the bHLH-MYC_N domain would cause *AtGL3* to lose its function in regulating NGT development, and therefore it can be hypothesized that motif 3 plays an important role in regulating trichome development [47]. In addition, motif 5 is not on any of the conserved structural domains but is unique to genes in two evolutionary branches that regulate trichome development, so it can be hypothesized that motif 5 also plays an important role in the regulation of trichome development in plants (Figure 1). *CsMYC2-CsMYC8* are in the GT-development regulating evolutionary branch with the same conserved motifs and domains as *SlMYC1*, *GoPGF*, and *GhCGF1*, suggesting that they may play a part in cucumber GT development. However, *CsMYC10-CsMYC13* are on a novel phylogenetic branch that has a conserved MYC structural domain but lacks several motifs compared to other genes.

### 2.2. Identification of Candidate CsMYCs in Cucumber GT Development

**Figure 2:**
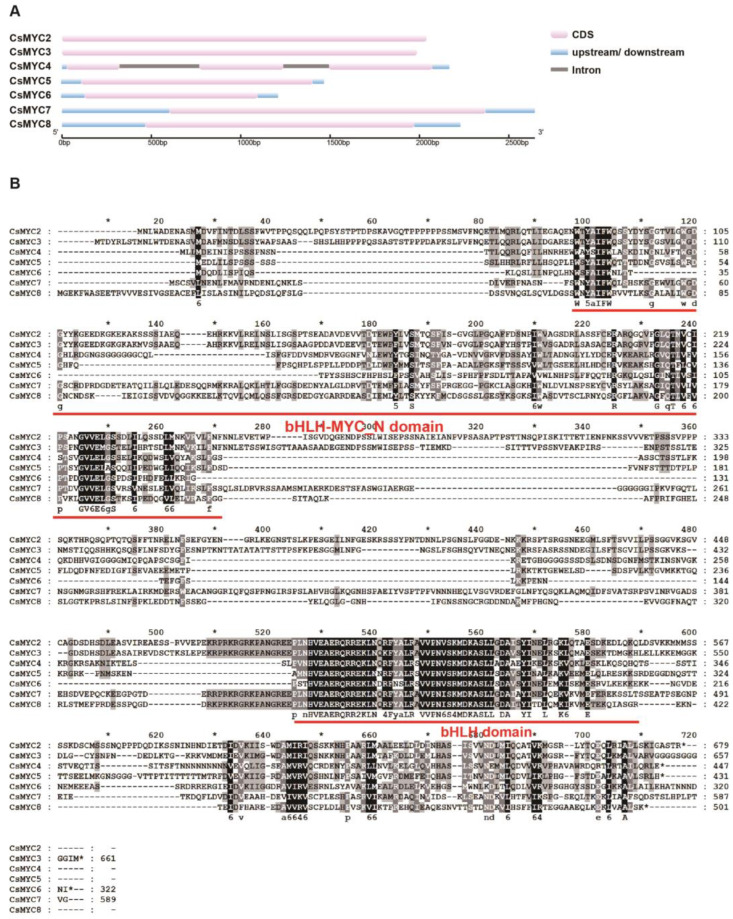
Bioinformatics analysis of *CsMYC2-CsMYC8*. (**A**) Gene structure analysis of *CsMYC2-CsMYC8* according to the cloning results. (**B**) Protein conserved domain alignment analysis of CsMYC2-CsMYC8.

To further investigate the role of the *CsMYCs* in cucumber GT development, we cloned the 7 *CsMYCs* (*CsMYC2-CsMYC8*) belonging to the group that regulates the GT development. Most of these *CsMYCs* contain only one exon, except *CsMYC4* (Figure 2A). According to the translation results based on the sequence of the cloned CDS, all of the candidate *CsMYCs* have a conserved bHLH-MYC_N domain and bHLH domain (Figure 2B).

**Figure 3:**
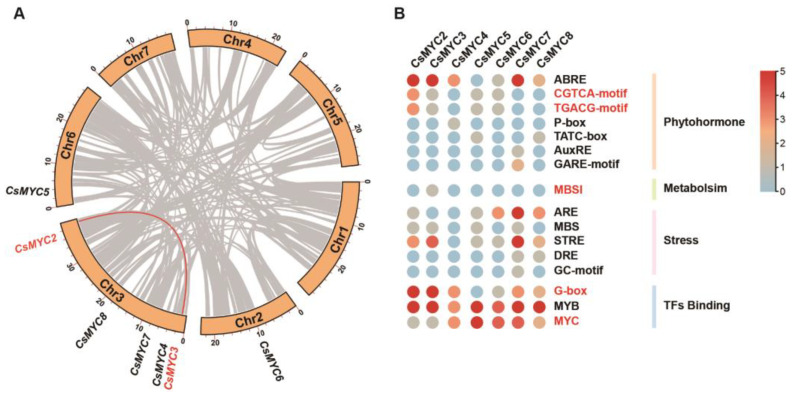
(**A**) Collinearity analysis of CsMYC2-CsMYC8 in cucumber. Grey lines show all gene duplications of the cucumber genome. Red lines show the duplicated genes in 7 candidates. (**B**) The *cis*-acting elements analysis of *CsMYC2-CsMYC8* promoters. The numbers of *cis*-acting elements are shown in a heatmap.

Co-linear fragments are large segments of homology within a single species resulting from genome duplication, chromosome duplication, or large segment duplication. Conserved gene sequencing within a homologous segment means that it may also be functionally conserved. Because of the high evolutionary homology among the seven candidates, we hypothesized that these genes might be subject to intraspecies gene duplication and therefore performed a collinearity analysis. The results show that in all gene duplications of the cucumber genome (grey line), there is only one pair of duplicated genes in seven candidates, which are *CsMYC2* and *CsMYC3* (red line) (Figure 3A). The 2000 bp upstream sequences of *CsMYC2-CsMYC8* were downloaded for *cis*-acting elements analysis and the number of *cis*-acting elements was calculated (Table S3). We found that the enriched *cis*-acting elements of the *CsMYC2-CsMYC8* promoter can be broadly classified into six categories: plant hormone response elements, plant metabolic pathway response elements, plant developmental response elements, plant stress response elements, plant light response elements, and other transcription factor binding elements (Table S3). Of these, *CsMYC2-CsMYC8* all contain a large number of MYB and MYC transcription factor binding elements (Figure 3B). Among the phytohormone response elements, *CsMYC2-CsMYC8* can be seen to respond to the regulation of various hormones, such as abs*cis*ic acid (ABRE), jasmonic acid (CGTCA-motif/TGACG-motif), auxin (AuxRE), and gibberellin (P-box/TATC-box/GARE-motif). Of these, abscisic acid and jasmonic acid have the largest number of response elements on the promoters of *CsMYC2-CsMYC8* (Figure 3B). In addition, *CsMYC2-CsMYC8* can respond to a variety of stress signals, such as drought (MBS) (Figure 3B, Table S3). Notably, among all seven MYC transcription factors, only the promoter of *CsMYC3* contains an element that responds to flavonoid metabolism (MBSI) (Figure 3B).

### 2.3. Candidate CsMYCs May Regulate the Formation of Cucumber GTs

**Figure 4:**
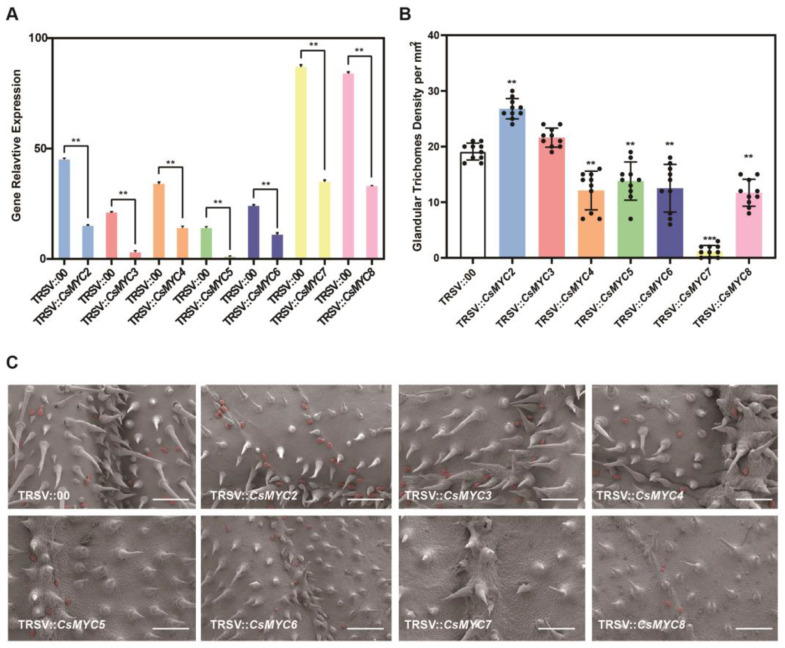
Gene function analysis of *CsMYC2-CsMYC8* in cucumber glandular trichomes. (**A**) Gene expression levels in the respective VIGS silenced plants. *CsTUA* served as the internal control. (**B**) Density (per mm^2^) of GTs in different VIGS silenced plants. Each scatter represents the mean of the density of each VIGS-positive plant. (**C**) Phenotypes of gene silenced plants on the first true leaf. The cucumber GT has been marked with a red color. Bars represent 500 μm. Error bars represent SD from three biological repeats. ** indicates *p*-value < 0.01. *** indicates *p*-value < 0.001.

We evaluated the possible roles of *CsMYC2-CsMYC8* in GT development through TRSV (tobacco ringspot virus)-based VIGS. For each of the seven genes, RT-qPCR reveals that the expression level in the VIGS plant is significantly lower than the control (Figure 4A), suggesting these genes are effectively silenced. The results of the VIGS suggest that almost all candidate *CsMYCs* are involved in the formation of GTs. TRSV::*CsMYC2* VIIGS plants show a significant increase in GT density (Figure 4B,C). TRSV::*CsMYC4*, TRSV::*CsMYC5*, TRSV::*CsMYC6*, TRSV::*CsMYC7*, TRSV::*CsMYC8* plants all show a significant decrease in GT density, in which TRSV::*CsMYC7* has the greatest decrease in GT density (Figure 4B,C). The GT density of TRSV::*CsMYC3* shows no change (Figure 4B,C). The phenotypes of VIGS imply that *CsMYC2* negatively regulates the formation of cucumber GTs, whereas *CsMYC4*, *CsMYC5*, *CsMYC6*, *CsMYC7*, and *CsMYC8* positively regulate the formation of cucumber GTs redundantly. VIGS of the cucumber *phytoene desaturase* gene (*CsPDS)* is used as the positive control (TRSV::*CsPDS*), which results in a photo-bleaching phenotype (Figure S2).

### 2.4. CsMYC2 and CsMYC7 Show Similar Expression Patterns in Cucumber

**Figure 5:**
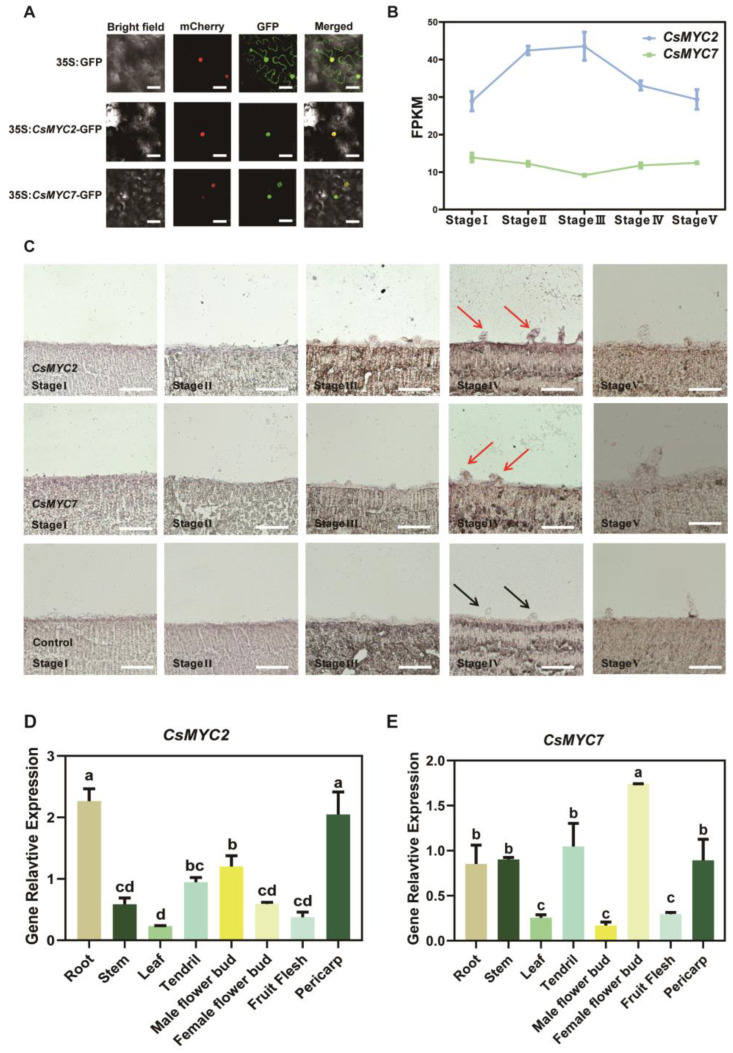
The expression patterns of *CsMYC2* and *CsMYC7.* (**A**) Subcellular localization indicating *CsMYC2* and *CsMYC7* fusion protein located in the nucleus of *N. benthamiana* leaves. Empty GFP driven by the super promoter was used as a control. The fluorescent signals of GFP channel indicate GFP position. The fluorescent signals of mCherry channel indicate mCherry-labeled nuclear marker position. Bars represent 50 μm. (**B**) FPKM of *CsMYC2* and *CsMYC7* from transcriptome data [10]. (**C**) In situ hybridization performed at five glandular trichome developmental stages of *CsMYC2* and *CsMYC7* in cucumber cotyledon. Black arrow shows cucumber GTs. Red arrow shows the expression of genes in cucumber GTs. Bars represent 1 mm. (**D**,**E**) Expression analyses of *CsMYC2* and *CsMYC7* in different cucumber organs. Error bars represent SD from three biological repeats. Different letter indicates statistically significant differences between groups.

Based on the observed phenotypes, we further analyzed the detailed expression patterns of *CsMYC2*, which have a unique negative regulatory role, and *CsMYC7*, which has a drastic phenotype. Subcellular localization results show that both *CsMYC2* and *CsMYC7* are localized in the nucleus (Figure 5A). Previous study has divided cucumber GT development into five stages, namely initiation stage (Stage I), first division stage (Stage II), glandular head transition stage (Stage III), glandular head formation stage (Stage IV), and active metabolism stage (Stage V). We next investigated the expression of these two genes in the transcriptomic data of the fifth stages and we found that they are both stably expressed during trichome development in cucumber (Figure 5B) [10]. Since these transcriptome data were sampled from the entire cotyledon and it is still unknown whether these two genes are expressed in GTs, we performed in situ hybridization. The results show that *CsMYC2* and *CsMYC7* are both specifically expressed in GTs at the fourth stage (Figure 5C). Expression analysis of *CsMYC2* and *CsMYC7* in different cucumber organs reveals that they are widely expressed in various tissue parts, with *CsMYC2* being highly expressed in the pericarp, root, and ovary, and *CsMYC7* being most highly expressed in the ovary, as well as in the pericarp, root, stem, and tendril (Figure 5D,E). These results suggest that both *CsMYC2* and *CsMYC7* have a range of functions in all parts of cucumber. They show similar expression patterns but antagonistic functions in GTs.

## 3. Discussion

MYC transcription factors are key components of the bHLH transcription factor family and have been extensively studied in maize, *Arabidopsis*, rice, and other crops [21,22,48]. However, comparatively little research has been conducted on MYC transcription factors in cucumber. In this study, we identified a total of 13 MYC transcription factors in cucumber, significantly more than in other crops, belonging to three distinct evolutionary branches, indicating a high degree of redundancy in MYC functions in cucumber (Table 1). Previous studies have demonstrated that MYC transcription factors play a role in the morphogenesis of both glandular and non-glandular trichomes [33,39,40,41,49]. Based on the important function of GTs, we focused our research on the role of MYC transcription factors in the regulation of GT development. Through a phylogenetic clustering analysis of previously reported MYC transcription factors, we found that the MYC transcription factors regulating the morphogenesis of GTs are on the same evolutionary branch, and seven of the 13 MYC transcription factors in cucumber belonging to this branch (Figure 1). This leads us to speculate that these seven genes are highly likely to regulate the development of GTs in cucumber. Previous studies have shown that a single amino acid change in the bHLH-MYC_N structural domain can alter the function of genes in regulating NGT development [47]. It remains to be seen if a similar change exists in the new branch that regulates GT development.

To further investigate the role of the *CsMYCs* in cucumber GT development, we cloned the seven *CsMYCs* (*CsMYC2-CsMYC8*) belonging to the group that regulates the GT development. Most of these *CsMYCs* contain only one exon, except *CsMYC4* (Figure 2A). Since *CsMYC4* is the most homologous gene for *GoPGF* in cotton and the cotton gland is a different lumenal structure from those of cucumber and tomato, we hypothesized that there may be some unique regulatory mechanism for *CsMYC4* [40].

Although the seven genes are highly homologous, gene duplication research found that there are only one possible pair of duplicated genes, which are *CsMYC2* and *CsMYC3* (Figure 3A). However, VIGS results suggest that *CsMYC2* can negatively regulate GT formation, while no function is observed for *CsMYC3* in cucumber GT development (Figure 4B,C). A similar homologous pair of genes is also present in tomato. In tomato, *SlMYC2*, a homolog of *SlMYC1* that regulates GT development, does not play a role in GT development but is involved in methyl jasmonate-induced tomato fruit resistance to pathogens [38,50]. Both *CsMYC2* and *CsMYC3* are the most homologous genes for *SlMYC1*. However, *CsMYC3* is the only one with a *cis*-acting element for responding to the biosynthesis pathway of flavonoids on its promoter (Figure 3B). GTs are the sites of secondary metabolite synthesis in plants, and *CsMYC3*, as the most homologous gene of *CsMYC2*, may be involved in the synthesis of secondary metabolites in GTs, which requires further investigation.

All seven candidate genes that potentially regulate the development of cucumber GTs were analyzed for *cis*-acting elements, and most of the candidate genes contain *cis*-acting elements in response to stress as well as plant hormones (Figure 3B). MYC transcription factors are known to play an important role in the jasmonic acid-induced stress response pathway [32,42,51,52]. The regulation of GT development by jasmonic acid has also been reported in *Artemisia annua* and tomato [53,54]. In cucumber, the role of MYC between GT development and the jasmonic acid response pathway needs to be further investigated. The genes that regulate the formation of GTs in response to stress signals also suggest that GTs, as ubiquitous structures on the plant epidermis, serve as the first line of defense for the plant. In addition, candidate *CsMYCs* have a large number of binding elements for MYC transcription factors on their promoters, suggesting that they may be regulated by homologous genes (Figure 3B).

Our results showed that *CsMYC2* negatively regulates the formation of GTs in cucumber, while *CsMYC4-CsMYC8* redundantly and positively regulate the formation of GTs, with *CsMYC7* being the primary effector gene (Figure 4). CsMYC2 is the first MYC transcription factor reported to negatively regulate GT formation, and its function deserves further exploration. In the previous transcriptome data, both *CsMYC2* and *CsMYC7* are stably expressed at five stages of multicellular trichome development without temporal variability (Figure 5B). However, the transcriptome sampling is not GTs-specific, the expression pattern was not representative of their function in cucumber GTs. Thus, in situ hybridization was performed and found that they are highly expressed in GTs at the fourth stage (Figure 5C). Combined with the VIGS results, it suggests that they are involved in different stages of glandular development, including not only the initiation differentiation from epidermal cells to trichome cells, but also the differentiation from trichome cells to glandular trichomes. Although we observed that *CsMYC2* and *CsMYC7* function in the leaf to regulate GT development, further expression patterns of these two genes reveal that they are expressed in various parts of the plant tissue, and the expression of them in leaf are relatively low. This result suggests that MYC transcription factors have powerful functions that are not limited to the regulation of GT formation. In addition, MYC transcription factors can form homopolymers or be regulated by each other, and we speculated that their expression may be influenced by each other (Figure 5D,E).

In *Arabidopsis*, the homologues of *CsMYC2* and *CsMYC7* are *AtMYC2* and *AtJAM2/AtbHLH13*, respectively. Previous studies have shown that AtJAM2/AtbHLH13 are transcriptional repressors and AtMYC2 is a transcriptional activator functioning antagonistically to bind the G-box of downstream genes to regulate the JA signaling pathway [27]. This study also seems to be consistent with our finding that *CsMYC2* and *CsMYC7* exhibit similar expression patterns but antagonistic functions, with CsMYC2 inhibiting GT formation while CsMYC7 positively regulating it (Figure 4 and Figure 5). The combined action of *CsMYC2* and *CsMYC7* helps to maintain the balance of GT formation, while the functional redundancy of *CsMYC7*, *CsMYC4*, *CsMYC5*, *CsMYC6*, and *CsMYC8* may ensure the stable formation of GTs. However, the VIGS phenotypes only represent the result of transient silencing. To gain a more comprehensive understanding of gene function, we need to construct stable, genetically modified knockout plants.

## 4. Materials and Methods

### 4.1. Gene Identification

The MYC transcription factors were firstly identified in the cucumber (Chinese Long) v2 genome (cucumber_ChineseLong_v2_pep downloaded from the cucurbit genomics website: http://cucurbitgenomics.org/ (accessed on 25 January 2023)) by their bHLH-MYC_N domain (PF14215) from the Pfam database (http://pfam-legacy.xfam.org/ (accessed on 25 January 2023)) using HMMER 3.0 software (E-value < 10^−5^) [55,56]. The NCBI-CDD database (NCBI Conserved Domains Database, https://www.ncbi.nlm.nih.gov/cdd (accessed on 25 January 2023)) and SMART database (Simple Modular Architecture Research Tool, http://smart.embl.de/smart/batch.pl (accessed on 5 March 2023)) were used to confirm the putative MYC proteins with both bHLH-MYC_N domain and bHLH domain [57,58]. The ExPASy website (https://web.expasy.org/compute_pi/ (accessed on 5 March 2023)) was used to predict the theoretical isoelectric point (pI) and molecular weight (Mw) of CsMYCs [59].

### 4.2. Phylogenetic Analysis

Multiple sequence alignment of all listed MYCs (Table S1) was carried out using ClustalW. The evolutionary history was inferred by using the Maximum Likelihood method based on the JTT matrix-based model [60]. Initial tree(s) for the heuristic search were obtained automatically by applying Neighbor-Join and BioNJ algorithms to a matrix of pairwise distances estimated using a JTT model, and then selecting the topology with superior log likelihood value. The analysis involved 22 amino acid sequences. All positions containing gaps and missing data were eliminated. There was a total of 226 positions in the final dataset. Evolutionary analyses were conducted in MEGA 7.0 [61]. The tree was further visualized by TBtools [62].

### 4.3. Gene Conserved Motif Identification

The gene conserved motif was analyzed by Multiple EM for Motif Elicitation (MEME) (https://meme-suite.org/meme/doc/meme.html?man_type=web (accessed on 5 March 2023)) [63]. The results were further visualized by TBtools [62].

### 4.4. Gene Cloning

The CDS and genome reference sequence of seven candidate *CsMYCs* were downloaded from cucurbit genomics database (http://cucurbitgenomics.org/ (accessed on 25 January 2023)). Cloning primers designed by PrimerPremier 5.0 software. Total DNA was extracted using a DNA extraction kit (Huayueyang, Beijing, China) and cDNA was reverse transcribed using a PrimeScript reagent Kit with gDNA Eraser (TaKaRa, Shiga, Japan) from total RNA extracted using an RNA extraction kit (Huayueyang, Beijing, China).

### 4.5. Gene Structure and Protein Conserved Domain Alignment Analysis

The gene structure was visualized by the Gene Structure Display Server (http://gsds.gao-lab.org/index.php (accessed on 25 January 2023)) [64]. Multiple sequence alignment was carried out using ClustalW and the output data were saved in FASTA format. Protein conserved domain alignment was analyzed by Genedoc 2.6.002 software.

### 4.6. Collinearity Analysis

Homologous gene pairs relationships of candidate *MYC* genes in cucumber were identified using Multiple Collinearity Scan toolkit (MCScanX) software with default parameters [65]. The results were visualized using advanced circus plot in TBtools [62].

### 4.7. Cis-Regulatory Elements Analysis

The promoter sequences (2000 bp upstream of ATG) of seven candidates were extracted from genome sequences. The cis-regulatory elements in promoter region were analyzed using the PlantCARE database (http://bioinformatics.psb.ugent.be/webtools/plantcare/html/ (accessed on 25 January 2023)) [66]. The heatmap of cis-regulatory elements analysis were generated by TBtools [62].

### 4.8. Plant Materials

The North China type (Chinese Long) cucumber inbred line Xintaimici (XTMC) was grown in a greenhouse of the China Agricultural University in Beijing. Pest control and water control followed standard practices. *Nicotiana benthamiana* plants were grown in a growth chamber set at 24 °C in a long-day condition (16-h light/8-h dark).

### 4.9. RNA Extraction and Real-Time Quantitative PCR (RT-qPCR) Analysis

Total RNA was extracted using an RNA extraction kit (Huayueyang, Beijing, China) and then reverse transcribed using a PrimeScript reagent Kit with gDNA Eraser (TaKaRa, Shiga, Japan). Subsequently, RT-qPCR was performed in 96-well plates with an ABI 7500 Real-Time PCR System (Applied Biosystems, Waltham, MA, USA) using SYBR Premix Ex Taq (TaKaRa, Shiga, Japan). For each sample, three biological and three technical replicates were conducted, with cucumber *CsTUA* (*α-tubulin*) as the reference. The gene-specific primers used for qPCR are listed in Table S4.

### 4.10. VIGS Assay and Phenotypic Observation

A modified tobacco ringspot virus (TRSV)-based virus induced gene silencing (VIGS) assay was performed to analyze the potential roles of candidate seven *CsMYCs* in cucumber [67]. In brief, a unique 300- to 500-bp CDS sequence for each candidate gene (primers are provided in Supplemental Table S4) was inserted into the SnaBI restriction site of pTRSV2 and then transformed into *Agrobacterium tumefaciens* GV3101. When the primary roots of germinating cucumber seeds reached ~1 cm, the seeds were infected with mixed pTRSV1 and pTRSV2 (containing different target fragments) by vacuum-infiltration under −900 kPa for 5 min. The seeds were then put on half MS solid medium with 100 μM acetosyringone until the agrobacterium was visible around the seeds. The seedlings were transplanted in half Hoagland solution for 15 d.

A total 10–12 VIGS positive plants were developed for each gene. The leaf blade with main vein of the first true leaf was then collected from each seedling. We sampled all VIGS plants to observe the phenotype under SEM. Three areas of each VIGS plant were sampled and three technical replicates of density observations were counted for each area, and finally the nine data were averaged to obtain the mean glandular trichome density of a single plant. Half of each sample was used for RT-qPCR to verify the gene expression level of gene transient silencing.

### 4.11. Scanning Electron Microscopy (SEM)

Samples were fixed with 2.5% (*v*/*v*) glutaraldehyde at 4 °C for ~24 h, washed with PBS (pH 7.2) three times, and post-fixed in 1% (*v*/*v*) OsO_4_. The samples were then dehydrated through an ethanol series (30, 50, 70, 80, 90, and 100%, three times), critical-point dried using a desiccator (HCP-2; Hitachi, Tokyo, Japan) and coated with gold palladium (EIKO IB-3). Images were taken with a Hitachi S-4700 scanning electron microscope using a 2-kV accelerating voltage.

### 4.12. Subcellular Localization

The full-length CDS without the stop codon of *CsMYC2* and *CsMYC7* was cloned into the pSUPER1300 vector and fused with the green fluorescent protein (GFP) to produce the CsMYC2-GFP and CsMYC7-GFP fusion protein. The empty pSUPER1300 vector was served as a control. The resultant constructs were introduced into the *Agrobacterium tumefaciens* strain GV3101. *Nicotiana benthamiana* leaves were co-transformed with the GFP-fusion construct and the nuclear location marker (mCherry). After 48 h infiltration, fluorescence signals were visualized at excitation/emission wavelength of 488/510 nm (GFP), 552/610 nm (mCherry), using a confocal laser scanning microscope (Leica SP8, Germany). The primer information is listed in Table S4.

### 4.13. In Situ Hybridization

The sampling method of multicellular trichome during development using cotyledon as material is described in a previous study [10]. Samples were fixed with 3.7% formalin–acetic acid–alcohol. In situ sense and antisense probes were transcribed with T7 RNA polymerase, respectively. Sample fixation, embedding, sectioning, and hybridization were conducted as previously described [68]. The primer information is listed in Table S4.

### 4.14. Accession Numbers

Sequence data from this article can be found in the Cucurbit Genomics Database (http://cucurbitgenomics.org/ (accessed on 25 January 2023)) and NCBI (https://www.ncbi.nlm.nih.gov/ (accessed on 25 January 2023)).

## 5. Conclusions

In conclusion, we conducted a genome-wide identification and analysis of MYC transcription factors in cucumber. Thirteen MYCs were identified in the *C. sativus* genome and their key structural features were compared to the previously reported MYCs from *Arabidopsis* and tomato to identify the *MYCs* involved in cucumber GT development. Seven candidate *MYCs* were analyzed and functionally characterized by VIGS, and it is found that *CsMYC2* is able to negatively regulate GT formation while *CsMYC4-CsMYC8* could positively regulate GT formation. The data obtained from this study provides new insights into the potential roles of *CsMYCs* in cucumber trichome development and establishes a basis for further research on cucumber GTs.

## Figures and Tables

**Table 1:** Detailed information about 13 predicted cucumber MYC genes.

Gene Name	Gene ID	Gene Position		CDS (bp)	Size (aa)	MW (kDa)	pI	Arabidopsis Homology
		Start	End (+/−)					
*CsMYC1*	Csa6G003480	351773	356445 (+)	1680	559	62,880.54	5.79	AT5G41315
*CsMYC2*	Csa3G902270	38986873	38988912 (+)	2040	679	74,590.96	5.11	AT1G32640
*CsMYC3*	Csa3G011620	1162977	1164962 (+)	1986	661	72,140.65	6.03	AT1G32640
*CsMYC4*	Csa3G002860	522560	524726 (−)	1344	447	49,382.41	8.66	AT4G00870
*CsMYC5*	Csa6G107910	7137128	7138598 (−)	1296	431	48,394.52	5.42	AT4G00870
*CsMYC6*	Csa2G080170	6627028	6628241 (+)	969	322	36,381.2	6.02	AT1G32640
*CsMYC7*	Csa3G119500	6736736	6739380 (+)	1770	589	65,256.14	5.73	AT1G01260
*CsMYC8*	Csa3G391380	19088549	19090777 (+)	1560	519	55,301.14	5.70	AT4G16430
*CsMYC9*	Csa5G601530	22011206	22013944 (+)	1422	473	53,390.76	5.70	AT2G16910
*CsMYC10*	Csa7G378380	13826607	13834438 (−)	2076	691	76,190.07	5.66	AT1G06150
*CsMYC11*	Csa3G733950	27943647	27948020 (+)	2085	694	78,486.44	5.24	AT2G27230
*CsMYC12*	Csa3G002970	600151	604757 (−)	2319	772	85,076.51	5.11	AT2G27230
*CsMYC13*	Csa1G632370	25233574	25240151 (−)	2880	959	104,477.47	6.40	AT2G27230

**Table 2:** Detailed information about MYC genes involved in plant trichome development.

Gene Name	Gene ID	Species	Function	References
*GoPGF*	Gh_A12G2172	*Gossypium hirsutum*	GT formation	[42]
*GhCGF1*	Gh_A11G0909	*Gossypium hirsutum*	GT formation	[43]
*SlMYC1*	Solyc08g005050	*Solanum lycopersicum*	Glandular cell division and expansion	[41]
*AtTT8*	AT4G09820	*Arabidopsis thaliana*	Marginal NGT development	[36]
*AtGL3*	AT5G41315	*Arabidopsis thaliana*	NGT formation	[37]
*AtEGL3*	AT1G63650	*Arabidopsis thaliana*	NGT formation	[38]
*AtMYC1*	AT4G00480	*Arabidopsis thaliana*	NGT formation	[35]
*GhDEL61*	LOC107904486	*Gossypium hirsutum*	NGT formation	[47]
*GhDEL65*	LOC107936704	*Gossypium hirsutum*	NGT formation	[48]

## Data Availability

The authors responsible for distribution of materials integral to the findings presented in this article are: Huazhong Ren (renhuazhong@cau.edu.cn) and Xingwang Liu (liuxw01@cau.edu.cn).

## Supplementary Materials

The following supporting information can be downloaded at: https://www.mdpi.com/article/10.3390/ijms24076435/s1.
