# Supplementary figures and images for: Identification and Functional Characterization of CsMYCs in Cucumber Glandular Trichome Development
Source: Int J Mol Sci. 2023 Mar 29;24(7):6435. doi: 10.3390/ijms24076435 (PMC10094329; doi:10.3390/ijms24076435)

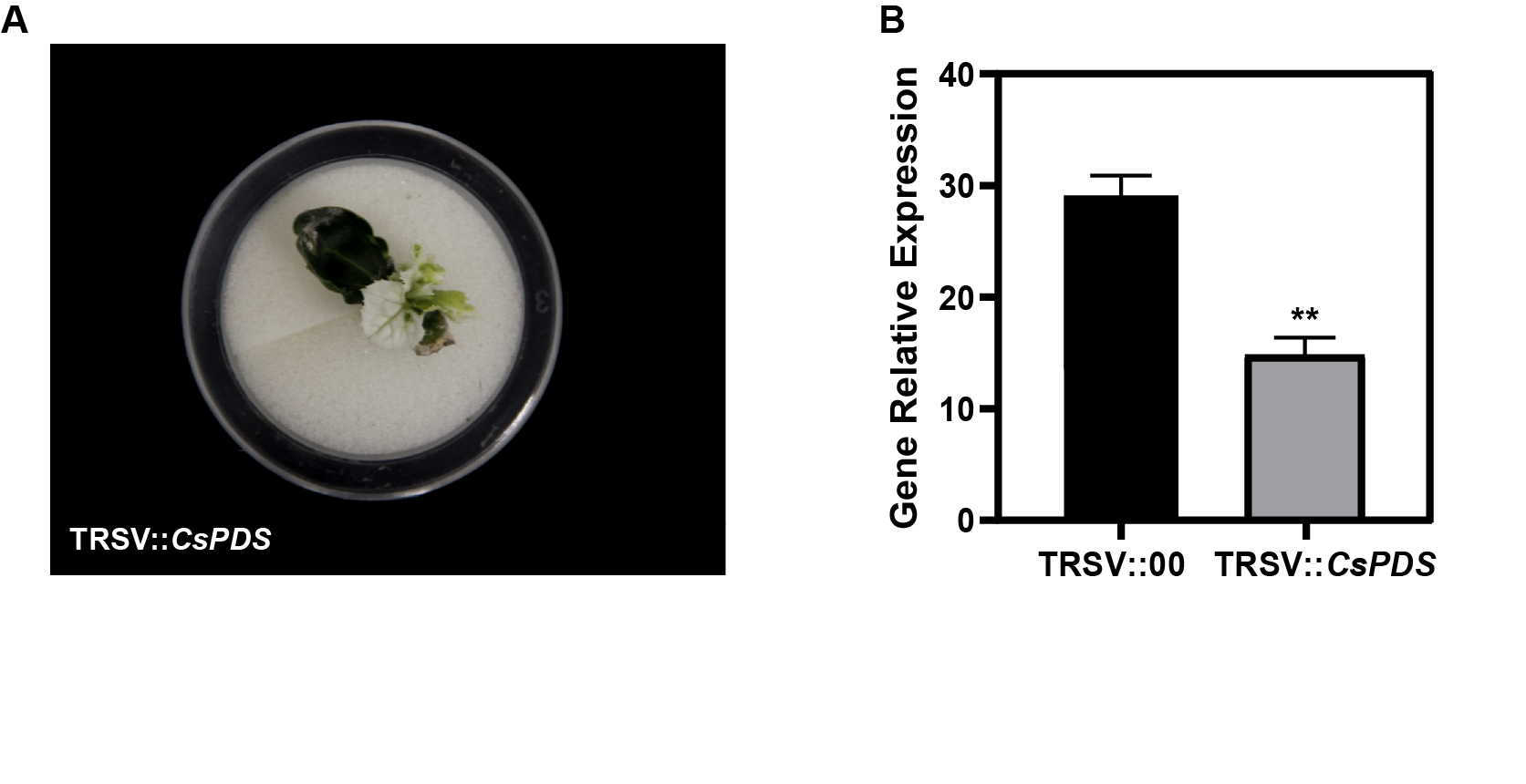

Supplement: Supplementary file 1 [file ijms-24-06435-s001.zip › Figure S2.tif]

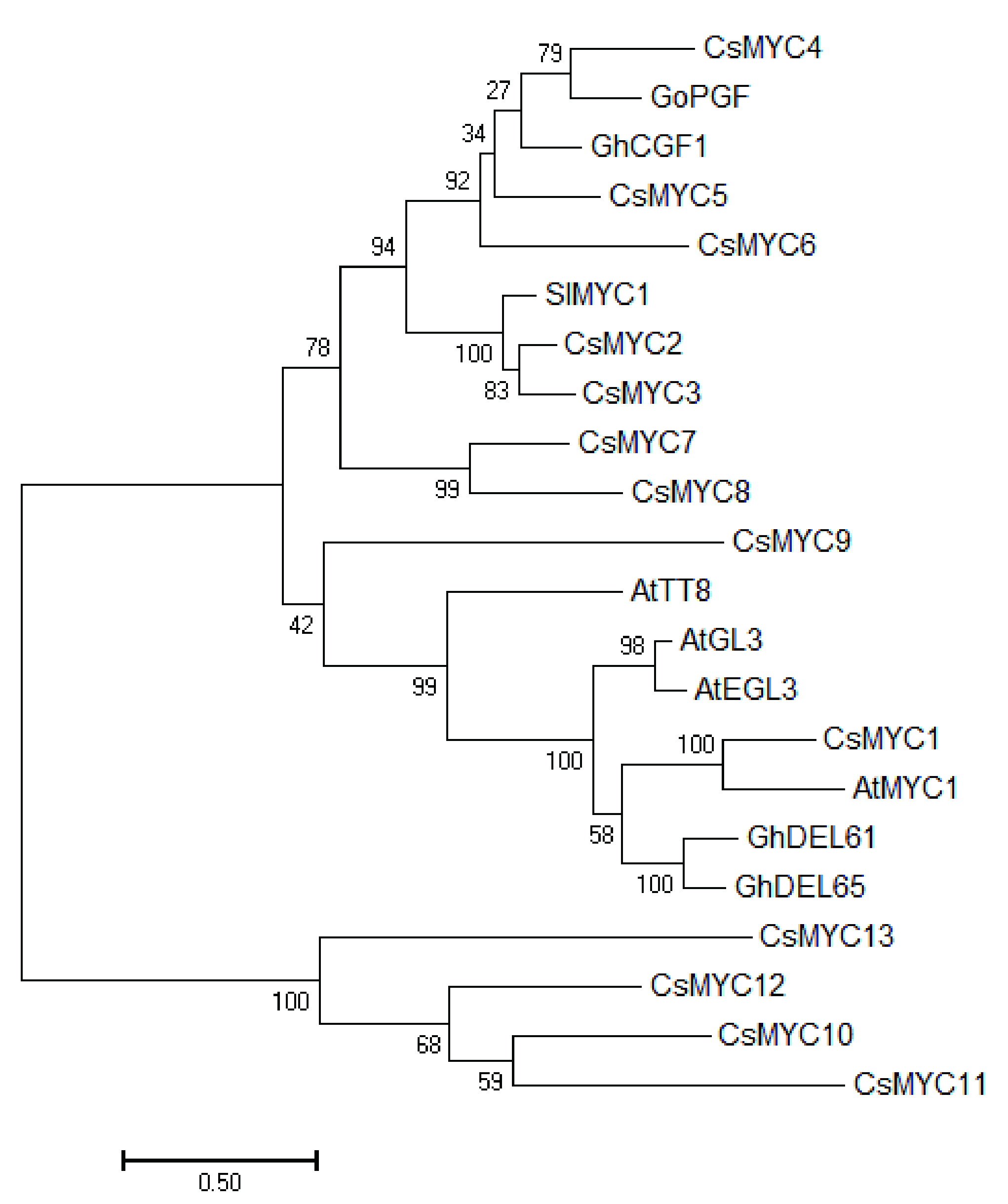

Supplement: Supplementary file 1 [file ijms-24-06435-s001.zip › Figure S1.tif]
